# Emotional affect in heart transplant candidates: a multicenter longitudinal study

**DOI:** 10.3389/fpsyg.2025.1608346

**Published:** 2025-08-06

**Authors:** Kelly M. Pennington, Abdelrahman Ahmed, Bradley K. Johnson, Daniel S. Yip, Roberto P. Benzo, Terry D. Schneekloth, Barry A. Boilson, Richard C. Daly, Cassie C. Kennedy

**Affiliations:** ^1^Division of Pulmonary and Critical Care Medicine, Mayo Clinic, Rochester, MN, United States; ^2^William J. von Liebig Center for Transplantation and Regenerative Medicine, Mayo Clinic, Rochester, MN, United States; ^3^Department of Health Services Research - Biomedical Statistics and Informatics, Mayo Clinic, Rochester, MN, United States; ^4^Department of Transplantation, Mayo Clinic Florida, Jacksonville, FL, United States; ^5^Department of Psychiatry, Mayo Clinic, Scottsdale, AZ, United States; ^6^Department of Cardiovascular Diseases, Mayo Clinic, Rochester, MN, United States; ^7^Department of Cardiovascular Surgery, Mayo Clinic, Rochester, MN, United States

**Keywords:** transplant, emotion, affect, PANAS, frailty

## Abstract

**Background:**

Emotional Affect reflects an individual’s emotional state and can be categorized as positive (PA) or negative (NA). We aimed to characterize affect in heart transplant candidates and evaluate its relationship with pre- and post-transplant psychological and clinical outcomes.

**Methods:**

Using the Positive and Negative Affect Schedule (PANAS), we surveyed adult heart transplant candidates across three transplant centers at baseline (waitlist enrollment), annually on the waitlist, and post-transplant. We assessed PA, NA, and the positivity ratio (PR; PA/NA) as potential predictors of waitlist mortality, post-transplant hospital length of stay, readmissions, and quality of life.

**Results:**

Among 194 participants, the majority were male (68.6%) and Caucasian (84.3%). Baseline PA (36.0 ± 7.8) and NA (17.9 ± 6.4) were comparable to population norms and remained stable over time. PR was low at baseline (2.3 ± 1.0) and decreased post-transplant (−0.3 ± 1.2; *p* = 0.03). PA decreased and NA increased post-transplant, but neither change was statistically significant. Affect was not associated with waitlist mortality, delisting, length of stay, or readmissions, but baseline PANAS scores correlated with multiple domains of post-transplant quality of life.

**Conclusion:**

Heart transplant candidates exhibit a suboptimal PR, which declines post-transplant, highlighting significant psychological stress. Pre-transplant PANAS scores correlated with post-transplant quality of life, suggesting a potential role for psychological screening and intervention in transplant care.

## Introduction

Heart transplant is the only definitive treatment for end-stage heart failure (HF) (Dokainish et al., 2017). Each year, there are approximately 7,300 patients awaiting heart transplant in the United States with 3,200 transplantations performed annually (Sandhu et al., 2019). It is estimated that the wait-list mortality is as high as 10% (Lund et al., 2017). Therefore, optimizing pre-transplant conditions of patients awaiting heart transplant is of paramount importance.

Patients awaiting heart transplant often face significant psychological stress, and emotional well-being has emerged as an important predictor of post-transplant outcomes in several organ groups (Chen et al., 2014). Frailty is an independent predictor of mortality in heart transplant candidates and recipients (Jha et al., 2018). Therefore, the International Society for Heart Lung Transplantation endorses assessing frailty in patients who are being evaluated for heart transplant (Mehra et al., 2016).

Psychological frailty refers to an individual’s cognitive and mood resilience in the presence of stressors. In recent years, there has been growing evidence that psychological frailty is equally as important as physical frailty in transplant outcomes. One study demonstrated that pre-operative depression and social isolation were associated with increased all-cause mortality following heart transplant (Spaderna et al., 2017). Moreover, depression is a predictor of poor compliance with medications after heart transplant and leads to an increased rate of re-hospitalization (Delibasic et al., 2017). Similarly, pre-transplant psychosocial vulnerability is associated with worse post-transplant psychosocial outcomes in liver, lung, and bone marrow transplant (Goetzmann et al., 2007). On the other hand, optimism has been shown to be associated with better survival in bone marrow transplant patients, particularly in the first 2 months following transplant (Lee et al., 2003). These factors are under studied as predictors of outcomes in heart transplant candidates.

Societal guidelines recommend psychosocial evaluation of all patients being considered for heart transplant (Mehra et al., 2016). This evaluation aims to detect and optimize psychosocial factors influencing patients’ health including cognitive function, adherence, psychopathology, social support and substance abuse (Bui et al., 2019). The methods used for this assessment are few and vary by center preference but include Psychosocial Assessment of Candidates for Transplantation (PACT), Stanford Integrated Psychosocial Assessment for Transplantation (SIPAT) and Transplant Evaluation Rating Scale (TERS) (Maldonado et al., 2012; Twillman et al., 1993). However, these assessment tools do not assess factors that can affect long-term psychological frailty and well-being like adjustment, resilience or optimism.

Emotional Affect refers to an individual’s emotional response or tone, and it may be categorized as positive or negative. Positive affect (PA) is the extent that an individual experiences pleasurable engagement with the environment (Crawford and Henry, 2004). In contrast, negative affect (NA) is the extent that an individual experiences pessimism, anger, unhappiness, nervousness and sadness (Watson et al., 1988). The Positive and Negative Affect Schedule (PANAS) is a validated 20-item scale that measures PA and NA. Additionally, the Positivity Ratio (PR)—calculated as the ratio of PA to NA—is used to distinguish individuals with optimal psychological functioning. A PR greater than 2.9 is associated with high emotional resilience and flourishing psychosocial health (Fredrickson and Losada, 2005). This threshold was originally proposed by Fredrickson and Losada (2005) in their work on emotional complexity and human flourishing. The PR has since been applied in a range of psychological and medical studies as an index of emotional resilience and adaptation, including in older adults and individuals with chronic illness (Diehl et al., 2011; Zautra et al., 2005). These applications support its relevance in evaluating psychological well-being among heart transplant candidates and recipients. Therefore, the PANAS may serve as a useful tool to assess emotional functioning and mental resilience. This study aims to: (1) describe affect in heart transplant candidates, (2) assess the change of affect in heart transplant candidates over time and following transplant, and (3) evaluate the relationship of pre-transplant affect with clinical and psychological outcomes before and after transplant.

## Methods

### Study design

This was a prospective, multicenter observational study conducted across three Mayo Clinic transplant centers (Rochester, MN; Jacksonville, FL; Scottsdale, AZ).

### Study population

Eligible participants were adults undergoing evaluation for their first heart transplant at one of three Mayo Clinic transplant centers. Patients with a prior solid organ transplant, multiorgan listing, or significant cognitive impairment were excluded. Eligible participants were approached for participation by mail (September 2015 to March 2019). Non-English-speaking patients and those without a domestic United States’ mailing address were excluded.

### Questionnaire administration

Demographic variables collected included age, sex, race/ethnicity, relationship status, and caregiver relationship to the patient. Questionnaires were mailed to study participants. After one-month, non-responders were sent a second questionnaire. One month following the second mailing, study coordinators contacted any non-responders by telephone to ensure questionnaire delivery and to invite participation. If interested, a third questionnaire was sent by mail or the participant completed the questionnaire by phone with the study coordinator. A small token of appreciation was included with the questionnaire (e.g., parking pass).

Questionnaires were re-administered annually by mail for patients remaining on the transplant waiting list. Additionally, a post-transplant questionnaire was collected 3 to 12 months following heart transplant. A secured, web-based Research Electronic Data Capture (REDCap^®^) database hosted by Mayo Clinic was used to store questionnaire data (Harris et al., 2009).

### Measures

#### Positive and Negative Affect Schedule (PANAS)

Affect was assessed using the 20-item PANAS, which measures two independent dimensions of affect. Ten items assess PA, characterized by high energy, enthusiasm, and alertness, with higher scores indicating greater PA (e.g., optimism; Cronbach’s *α* = 0.89) (Crawford and Henry, 2004; Fredrickson and Losada, 2005). The remaining 10 items assess NA, reflecting distress and aversive emotions, with higher scores indicating greater NA (e.g., pessimism; Cronbach’s α = 0.85) (Crawford and Henry, 2004; Fredrickson and Losada, 2005). It has been postulated that a ratio of positive to negative affect, also known as positivity ratio (PR), ≥2.9 can differentiate individuals with flourishing mental health and emotional resilience (Crawford and Henry, 2004; Fredrickson and Losada, 2005; Watson et al., 1988). The minimal clinically important difference (MCID), defined as one-half of the standard deviation, is 3.8 for PA and 3.0 for NA (Pennington et al., 2020).

#### Kansas City Cardiomyopathy Questionnaire (KCCQ)

The Kansas City Cardiomyopathy Questionnaire (KCCQ) assesses health-related quality of life (QOL) in patients with heart failure. This 23-question tool quantifies the following domains: physical limitations, symptoms (including frequency, severity and change over time), self-efficacy, and social interference (Green et al., 2000). Scores are transformed to a 0–100 scale with 100 representing the least severe symptoms and 0 representing the most severe symptoms. Internal consistency has been validated with overall Cronbach α 0.95 (Masterson Creber et al., 2012; Mishra et al., 2015). The MCID for improvement in HF patients using KCCQ is less than 5 points (Butler et al., 2020).

### Clinical data abstraction

Questionnaires included basic demographic information and relationship of primary caregiver to the patient. Additional data regarding participant demographics, diagnosis, transplant listing, length of stay, acute rejection, and survival outcome were abstracted from the participant electronic medical record. All outcomes were censored as of August 21, 2019. Chart review was performed by two trained study staff members and independently verified for accuracy.

### Objectives and outcomes

Our primary objective was to characterize positive and negative affect in heart transplant candidates, including changes over time and following transplant. Our secondary objective was to evaluate PANAS scores as potential predictors of transplant-related outcomes. The primary predictors were PA, NA, and PR at study enrollment.

Our primary outcome was a composite of death on the waiting list or delisting due to deterioration. Secondary outcomes included pre- and post-transplant health-related QOL, post-transplant mortality, transplant procedure hospital length of stay (LOS), and post-transplant readmissions and time to readmission. Specific reasons for readmission (e.g., scheduled follow-up, acute rejection, infection) were not consistently documented across sites and were therefore not included in the analysis.

### Statistical analysis

PANAS scores were calculated at baseline (study enrollment), annually on the waitlist, and post-transplant and are reported as mean (± standard deviation). PANAS single-item responses are described as percentages, categorized as responses of one to two (“not at all” to “a little”) versus three or more (“moderately” to “extremely”).

Group comparisons were performed using chi-square or Kruskal–Wallis tests. Changes in PA, NA, and PR over time were analyzed using a one-sample, paired *t*-test. The strength of relationships between PANAS scores and continuous outcomes (KCCQ scores, hospital LOS, and time to readmission) was assessed using Pearson’s correlation coefficient (*r*).

Associations with death, delisting, and readmission were evaluated using Cox proportional hazard ratios (HR). Univariate logistic regression was used to assess the association of baseline PA, NA, and PR with outcomes of interest. Models were adjusted for time on the waitlist, sex, and age.

All analyses were conducted using SAS version 9.4 (SAS Institute Inc.; Cary, NC, United States). A *p*-value ≤0.05 was considered significant, and no corrections were made for multiple comparisons.

### Missing data

Participants were only included in the analysis if baseline PANAS was completed. Partially completed questionnaires were not analyzed.

### Ethical considerations

This study was approved by the Mayo Clinic Institutional Review Board (IRB# 15–00537) and conducted in accordance with the Declaration of Helsinki. Written informed consent was obtained from all participants prior to enrollment.

## Results

### Participants

**Figure 1:**
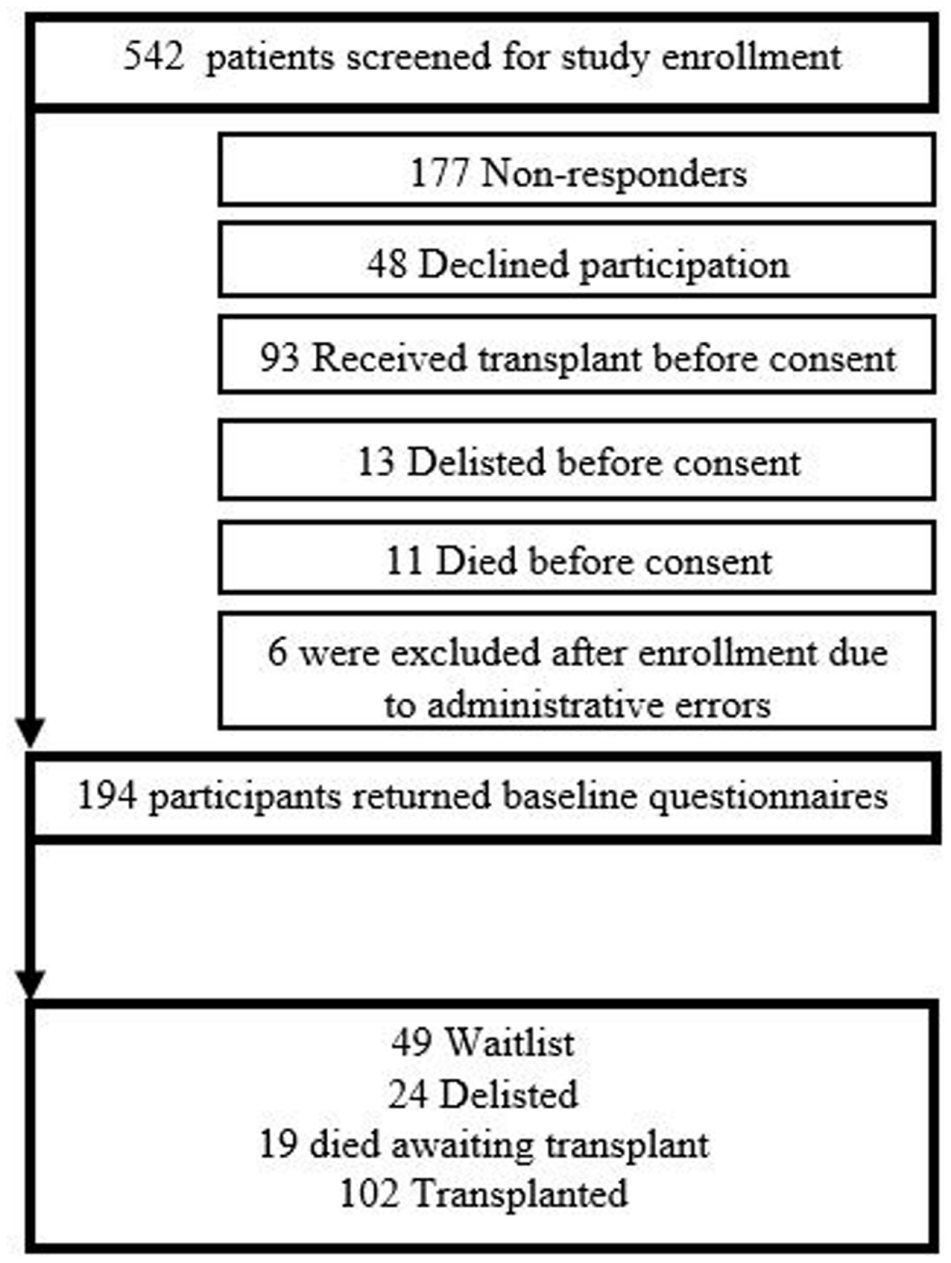
Flowchart of study enrollment and participant outcomes.

Baseline questionnaires were completed by 194 of the 371 (52.3%) consented candidates (*n* = 194) (Figure 1). The median time on the waitlist prior to enrollment was 2.7 months (interquartile range [IQR] 1.2 to 9.7). Most participants were male (68.6%) and Caucasian (84.3%), with a median age of 57.4 years (IQR 48.5 to 62.4) (Table 1). Approximately one-third (61, 31.4%) of patients had a left ventricular assist device (LVAD). The majority of candidates were married (73.2%), with their spouse identified as the primary caregiver (69.9%).

**Table 1:** Baseline demographic and clinical characteristics of heart transplant candidates who completed the Positive and Negative Affect Schedule (PANAS) questionnaire at enrollment (*n* = 194).

	Median (IQR) or *n* (%)
*n*	194
Age (yrs)	*57.4 (48.5, 62.4)*
Gender	
Male	133 (68.6)
Race	
Caucasian	164 (84.5)
Other	30 (15.4)
BMI	
Underweight (<18.5)	1 (0.5)
Normal weight (18.5–24.9)	41 (21.1)
Overweight (25.0–29.9)	76 (39.2)
Obese (>29.9)	76 (39.2)
Marital Status	
Never married	28 (14.4)
Married	142 (73.2)
Widowed	3 (1.5)
Separated or divorced	21 (10.8)
Education	
Have not graduated high school	11 (5.7)
High school graduate	34 (17.5)
Trade school or some college	76 (39.2)
Bachelor’s degree	38 (19.6)
Advanced degree	34 (17.5)
Unknown	1 (0.5)
Primary caregiver	
Parent	28 (14.4)
Spouse	135 (69.6)
Significant Other	10 (5.2)
Other	20 (10.3)
Unknown	1 (0.5)
Reason for Transplant	
Dilated Cardiomyopathy	79 (40.7)
Ischemic Cardiomyopathy	51 (26.3)
Congenital Heart Defects	14 (7.2)
Valvular Heart Disease	7 (3.6)
Infiltrative Cardiomyopathy	11 (5.7)
Hypertrophic Cardiomyopathy	9 (4.6)
Postpartum Cardiomyopathy	4 (2.1)
Retransplant	5 (2.6)
Other	14 (7.2)

Data are presented as median (interquartile range) for continuous variables [italicized] or *n* (%) for categorical variables.

BMI, body mass index; yrs, years.

Of the 194 participants, 102 received a transplant, 49 remained on the waitlist, 19 died awaiting transplant, and 24 were removed from the waitlist due to clinical deterioration (Figure 1). Among the 102 transplanted participants, 4 died prior to index hospital discharge. The median length of stay (LOS) was 15.0 days (IQR 10.0–22.5). Post-transplant questionnaires were completed by 67 (65.7%) of 102 transplanted participants. Among the 98 surviving transplanted patients, 61 (62.2%) experienced a readmission post-transplant, with a median time to readmission of 68.0 days (IQR 29.3–114.3).

#### PANAS

At baseline, the mean PA score was 36.0 ± 7.8, and the mean NA score was 17.9 ± 6.4 (*n* = 194). The mean PR at baseline was 2.3 ± 1.0, with the majority of participants (143, 73.5%) falling below the optimal functioning threshold of 2.9.

At baseline, participants’ single-item PANAS responses revealed that more than one-quarter reported feeling moderately to extremely distressed (27.9%), scared (30.8%), irritable (29.9%), nervous (36.9%), and/or afraid (25.2%) (Table 2). Baseline PA did not differ by sex, age, diagnosis, waitlist duration, or marital status.

**Table 2:** Percentage of heart transplant candidates identifying with positive and negative emotions on the Positive and Negative Affect Schedule (PANAS) at baseline assessment (*n* = 194).

Emotion		Degree of identification with stated emotion		
		Not at all to a little (%)	Moderately (%)	Quite a bit to extremely (%)
Positive emotions	1. Interested	6.0	22.2	70.2
	2. Excited	37.1	32.0	30.0
	3. Strong	18.8	27.4	52.8
	4. Enthusiastic	24.4	31.5	42.6
	5. Proud	16.7	29.8	52.1
	6. Alert	9.1	17.7	71.2
	7. Inspired	22.8	23.2	52.5
	8. Determined	4.5	17.7	76.3
	9. Attentive	11.1	26.3	60.7
	10. Active	18.8	31.5	48.3
Negative emotions	11. Distressed	71.5	20.2	7.7
	12. Upset	80.2	14.2	5.1
	13. Guilty	86.9	9.6	2.0
	14. Scared	67.7	17.2	13.6
	15. Hostile	91.9	5.1	1.5
	16. Irritable	69.0	21.8	8.1
	17. Ashamed	91.4	5.6	1.5
	18. Nervous	62.1	25.3	11.6
	19. Jittery	75.2	14.7	8.6
	20. Afraid	73.7	12.1	13.1

Responses are categorized as “Not at all to a little,” “Moderately,” and “Quite a bit to Extremely.” Data are presented as percentages. PANAS = Positive and Negative Affect Schedule.

While on the waitlist (*n* = 49), PA (mean change +1.4 ± 6.8; *p* = 0.69), NA (+0.4 ± 5.6; *p* = 0.89), and PR (+0.1 ± 0.96; *p* = 0.74) did not change significantly over time. However, following transplant (*n* = 67), PR decreased significantly (−0.3 ± 1.2; *p* = 0.03). PA also decreased (−0.9 ± 8.2; *p* = 0.35), while NA increased (+1.4 ± 7.0; *p* = 0.11), though neither change reached statistical significance.

We also stratified post-transplant PANAS scores by time of survey administration (3–6 months vs. 7–12 months post-transplant). There were no statistically significant differences in positive affect, negative affect, or the positivity ratio between the two timeframes (all *p* > 0.70), suggesting relative stability in affect during the first year after transplant.

### Outcomes

#### Pre-transplant

The PA, NA, and PR were not associated with death or delisting. The hazard ratio (HR) for death or delisting was 1.00 (95% CI: 0.97–1.04, *p* = 0.91) for PA, 0.98 (95% CI: 0.94–1.02, *p* = 0.38) for NA, and 1.58 (95% CI: 0.82–3.07, *p* = 0.17) for PR < 2.9.

These associations remained non-significant after adjustment for sex, age, and time on the waitlist (PA: HR 0.99, 95% CI: 0.97–1.04, *p* = 0.95; NA: HR 0.99, 95% CI: 0.95–1.05, *p* = 0.96; PR: HR 1.18, 95% CI: 0.59–2.34, *p* = 0.64).

#### Post-transplant

Baseline PA, NA, and PR correlated with post-transplant KCCQ scores, including overall summary, total symptom, clinical summary, symptom burden, and QOL scores (Table 3). However, baseline PANAS scores were not associated with hospital LOS (PA: *r* = 0.02, *p* = 0.85; NA: *r* = 0.09, *p* = 0.40; PR: *r* = −0.09, *p* = 0.38). Affect also did not predict readmission (PA: HR 1.02, *p* = 0.21; NA: HR 0.99, *p* = 0.79; PR: HR 1.26, *p* = 0.51) or time to readmission (PA: *r* = −0.02, *p* = 0.86; NA: *r* = 0.03, *p* = 0.82; PR: *r* = −0.07, *p* = 0.59).

**Table 3:** Association between pre-transplant affect scores and post-transplant Kansas City Cardiomyopathy Questionnaire (KCCQ) sub-scores (*n* = 67).

Variable	PA Coef	*P*-value	NA Coef	*P*-value	PR Coef	*p*-value
Physical Limitation Score	**0.99**	0.001*	−0.42	0.2	4.09	0.07
Symptom Stability Score	0.45	0.21	−0.13	0.75	1.52	0.56
Symptom Frequency Score	**0.84**	0.003*	−0.52	0.11	4.63	0.03*
Symptom Burden Score	**0.95**	0.001*	**−0.74**	0.03*	**7.35**	<0.001*
Self-Efficacy Score	0.35	0.05*	−**0.45**	0.03*	2.44	0.06
QOL Score	**1.29**	<0.001*	**−1.33**	<0.001*	**9.85**	<0.001*
Social Limitation Score	0.75	0.07	−0.46	0.31	3.98	0.17
Total Symptom Score	**0.90**	0.001*	**−0.63**	0.04*	**5.99**	0.002*
Overall Summary Score	**0.97**	<0.001*	**−0.71**	0.02*	**6.00**	0.001*
Clinical Summary Score	**0.93**	<0.001*	−0.52	0.07	**4.99**	0.008*

Results shown as unadjusted linear regression coefficients (Coef) with associated *p*-values for each PANAS domain: Positive Affect (PA), Negative Affect (NA), and Positivity Ratio (PR).

Coef, coefficient; NA, negative affect; PA, positive affect; PR, positivity ratio; QOL, quality of life.

*Denotes statistically significant result. Bolded values denote statistically significant result.

## Discussion

Our primary findings can be summarized as follows: (1) the majority of waitlisted heart transplant patients have a PR below the threshold for optimal emotional well-being; (2) PR decreases following heart transplant; (3) many heart transplant candidates report feeling distressed or scared; (4) baseline PANAS scores predict post-transplant QOL; and (5) affect is not associated with key physical transplant outcomes.

Interestingly, baseline PA and NA scores in our cohort were comparable to values reported in healthy college students (PA: 33.3 ± 7.2, NA: 17.4 ± 6.2) (Crawford and Henry, 2004). However, the mean PR in our cohort was 2.3—lower than the optimal threshold of 2.9 and only modestly higher than the average PR of healthy young adults (≈1.9–2.1) (Fredrickson and Losada, 2005). This suggests that although transplant candidates may not have severely elevated NA or suppressed PA, the combination of slightly elevated negative emotions with only modest positive engagement results in a suboptimal emotional balance. While standardized scoring approaches such as the sten scale offer a useful framework for classifying affective functioning, we chose to use raw PANAS scores and the positivity ratio in this analysis to align with validated methods commonly reported in transplant and psychosocial research. Norm-based transformations would require population-specific reference data stratified by clinical characteristics, which were not uniformly available across our sites. Future studies may benefit from incorporating normalized affect scores to enable trajectory-based categorization (e.g., movement from low to moderate affect) and comparison with external populations. The PR represents the balance of positive to negative emotions over time and is a predictor of psychological well-being (Fredrickson and Losada, 2005). In our cohort, the mean PR was 2.3, below the optimal threshold of >2.9, suggesting that heart transplant candidates may be less emotionally equipped to adapt to novel or stressful situations (Fredrickson and Losada, 2005). The further decline in PR post-transplant may reflect the psychological toll of transplantation or persistent gaps in psychological support following transplant. Several mechanisms may underlie the observed shifts in PA and NA following transplantation. The early post-transplant period is often marked by physical debility, high treatment burden, and uncertainty around graft function, which may contribute to elevated NA. Concurrently, immunosuppressive medications—particularly corticosteroids—can affect mood regulation and may exacerbate irritability, anxiety, or depressive symptoms. On the other hand, the decrease in PA may reflect diminished energy, loss of autonomy, or unmet expectations regarding recovery. Social isolation, changes in caregiver dynamics, or employment disruptions may also influence emotional states. These multifactorial contributors highlight the need for integrated psychosocial care during the transplant recovery period. Our findings align with prior research demonstrating increased depression and psychological distress after organ transplantation (Dew et al., 2012; Stilley et al., 1999). This is clinically significant, as post-transplant psychiatric conditions, particularly depression, have been associated with increased mortality (Dew et al., 1999).

Moreover, over a quarter of participants reported feeling moderately to extremely distressed (28%), scared (30.8%), irritable (30%), nervous (36.9%), or afraid (25.3%), consistent with prior research on high rates of anxiety and depression in patients awaiting heart transplant. For example, Schneekloth et al. (2019) found that 17 and 27% of waitlisted heart transplant patients experienced anxiety and depression episodes, respectively, in the year preceding transplant.

At baseline, a substantial proportion of participants reported experiencing distressing emotions: 28% felt moderately to extremely distressed, 30.8% scared, 30% irritable, 36.9% nervous, and 25.3% afraid. At the same time, many participants endorsed strong positive emotions: 76.3% reported feeling determined, 71.2% alert, 70.2% interested, 60.7% attentive, and 52.8% strong. This mixed emotional profile underscores the psychological complexity of transplant candidacy—patients frequently experience concurrent feelings of purpose and resilience alongside fear, anxiety, or uncertainty.

Affect was not associated with LOS, readmissions, or mortality. Prior studies have linked pre-transplant psychological factors—particularly depression—to longer hospital stays. For example, Rogal et al. (2016) found that depression in liver transplant candidates was associated with prolonged LOS during the transplant hospitalization. Similarly, patients with pre-existing mood or anxiety disorders had longer LOS during stem cell transplantation (Prieto et al., 2002). We previously demonstrated that lung transplant candidates with higher negative affect had an increased risk of waitlist mortality (Pennington et al., 2020).

Our findings also suggest that PANAS scores can predict post-transplant QOL, as they correlated closely with post-transplant KCCQ scores. QOL in transplant recipients is shaped by interactions between physiologic, social, and psychological factors (Angermann et al., 1992). Additional factors such as employment status, caregiver burden, or return-to-work capability were not captured in this study but may meaningfully influence emotional recovery and QOL. Grady et al. (1999) identified nine key predictors of post-transplant QOL at 1 year, including lower stress, better adherence to the transplant regimen, effective coping strategies, fewer functional limitations, lower symptom burden, older age, fewer complications, access to helpful healthcare information, and positive health perceptions. While some of these factors emerge only after transplant, our findings highlight the potential of pre-transplant PANAS scores to identify at-risk patients. Targeting these vulnerable individuals with pre- or post- transplant psychological interventions may enhance coping skills and improve post-transplant QOL. Furthermore, stratification by timing of post-transplant survey (3–6 vs. 7–12 months) did not reveal significant differences in affect, indicating that psychological recovery may stabilize early after transplant in many patients. While we did not conduct stratified analyses by baseline PR category, future studies with larger longitudinal samples may help determine whether individuals with higher pre-transplant positivity ratios—indicative of flourishing mental health—demonstrate more resilient emotional trajectories after transplantation.

Similar associations between psychosocial vulnerability and outcomes have been observed in other transplant populations. For example, in liver transplant candidates, pre-transplant depression has been linked to increased post-operative complications, longer hospital stays, and reduced survival (Rogal et al., 2016) likewise, kidney transplant recipients with baseline depressive symptoms report lower post-transplant quality of life and increased healthcare utilization (Griva et al., 2014; Palmer et al., 2013; Rogal et al., 2016). These findings underscore the generalizability of our results and the importance of addressing psychological well-being across transplant populations. Several evidence-based interventions have shown potential for improving post-transplant QOL. Early identification of psychological distress through routine screening tools such as the PANAS or PHQ-9 can help flag at-risk patients. Cognitive behavioral therapy (CBT) has been shown to reduce anxiety and depressive symptoms in solid organ transplant recipients (Epstein et al., 2019; Rodrigue et al., 2011; Rogal et al., 2011). Additionally, structured peer mentoring, mindfulness-based stress reduction, and multidisciplinary care models that integrate mental health providers into transplant teams have been associated with improved coping and adherence (Chida and Steptoe, 2008). Further work is needed to evaluate the scalability and long-term effectiveness of these strategies in heart transplant populations.

### Limitations

Our study had a relatively small sample size, which limited our ability to fully assess the impact of affect on transplant outcomes. Additionally, the follow-up period post-transplant was short, potentially underestimating the extent of affect changes over time. The low number of post-transplant deaths likely resulted in insufficient power to detect an association between affect and post-transplant mortality.

Although the sickest heart transplant candidates were not explicitly excluded, their participation was likely limited due to reduced capacity to complete surveys and shorter wait times driven by increased transplant urgency. Despite these limitations, our study provides valuable insights into the role of affect in heart transplant candidates. A larger, prospective study with extended follow-up is needed to further define the predictive value of affect in this population and confirm our findings. Finally, although we examined the association between pre-transplant affect and post-transplant QOL using the KCCQ, we did not collect KCCQ data pre-transplant and were therefore unable to assess baseline QOL or explore the direct association between pre-transplant affect and contemporaneous QOL. Future studies incorporating both pre- and post-transplant QOL assessments may help further elucidate this relationship.

## Conclusion

### Pre-transplant

Heart transplant candidates demonstrated a suboptimal positivity ratio at baseline, despite otherwise average affect scores, suggesting underlying emotional strain during the evaluation and waitlist period. PANAS scores obtained at this stage were significantly associated with post-transplant QOL, supporting their potential value as a screening tool.

### Post-transplant

Affect scores did not significantly improve after transplant and, in some cases, declined, underscoring the need for continued psychological support. These findings suggest that the stressors of recovery, medication side effects, and lifestyle disruption may offset expected emotional relief.

### Implications

Incorporating emotional health assessments into standard transplant care may help identify vulnerable patients and improve long-term recovery through timely intervention. Future studies should explore broader psychosocial factors and test scalable interventions aimed at promoting emotional resilience throughout the transplant journey.

## Data Availability

The raw data supporting the conclusions of this article will be made available by the authors, without undue reservation.
